# Anthocyanin supplementation in adults at risk for dementia: a randomized controlled trial on its cardiometabolic and anti-inflammatory biomarker effects

**DOI:** 10.1007/s11357-025-01669-8

**Published:** 2025-05-02

**Authors:** Miguel German Borda, Robinson Ramírez-Vélez, Felipe Botero-Rodriguez, Jonathan Patricio-Baldera, Chiara de Lucia, Ilaria Pola, George E. Barreto, Khadija Khalifa, Anne Katrine Bergland, Miia Kivipelto, Tommy Cederholm, Henrik Zetterberg, Nicholas J. Ashton, Clive Ballard, Richard Siow, Dag Aarsland

**Affiliations:** 1 Centre for Age-Related Medicine (SESAM), Stavanger University Hospital, Helse Stavanger HF, Postboks 8100, 4068, Stavanger, Norway; 2 Department of Neurology, Clínica Universidad de Navarra, Pamplona, Spain; 3 Centro de Investigación en Ciencias de La Salud (CICSA), FCS, Universidad Anáhuac México, Huixquilucan Edo. de México, México; 4 Navarrabiomed, IdiSNA, Hospital Universitario de Navarra (CHN), Universidad Pública de Navarra (UPNA), Pamplona, Spain; 5 SynaptIA – Inteligencia artificial para la investigación en salud mental, Bogotá, Colombia; 6 Centro de Memoria y Cognición Intellectus, Hospital Universitario San Ignacio, Bogotá, Colombia; 7 Instituto de Investigación en Salud, Facultad de Ciencias de La Salud de La Universidad Autónoma de Santo Domingo, Santo Domingo, Dominican Republic; 8 Centre for Healthy Brain Ageing, Institute of Psychiatry, Psychology, and Neuroscience, King’s College London, London, UK; 9 Department of Psychiatry and Neurochemistry, Institute of Neuroscience and Physiology, the Sahlgrenska Academy, University of Gothenburg, Mölndal, Sweden; 10 Department of Biological Sciences, University of Limerick, Limerick, Ireland; 11 Department of Clinical Medicine, University of Bergen, Bergen, Norway; 12 Division of Clinical Geriatrics, Center for Alzheimer Research, Department of Neurobiology, Care Sciences and Society, Karolinska Institutet, Stockholm, Sweden; 13 Department of Public Health and Caring Sciences, Clinical Nutrition and Metabolism, Uppsala University, 62167 Uppsala, Sweden; 14 Theme Inflammation & Ageing, Karolinska University Hospital, Stockholm, Sweden; 15 Clinical Neurochemistry Laboratory, Sahlgrenska University Hospital, Mölndal, Sweden; 16 Department of Neurodegenerative Disease, UCL Institute of Neurology, Queen Square, London, UK; 17 UK, Dementia Research Institute at UCL, London, UK; 18 Hong Kong Center for Neurodegenerative Diseases, Clear Water Bay, Hong Kong, China; 19 Wisconsin Alzheimer’s Disease Research Center, School of Medicine and Public Health, University of Wisconsin, University of Wisconsin-Madison, Madison, WI USA; 20 Banner Alzheimer’s Institute, University of Arizona, Phoenix, AZ USA; 21 Medical School, University of Exeter, University of Exeter, Exeter, UK; 22 School of Cardiovascular and Metabolic Medicine & Sciences, Faculty of Life Sciences & Medicine, British Heart Foundation Centre of Research Excellence, King’s College London, London, UK

**Keywords:** Inflammation, Inflammation mediators, Cytokines, Anthocyanins, Clinical trial, Aging

## Abstract

Anthocyanins are dietary flavonoids shown to have a therapeutic capacity to mitigate inflammation and oxidative stress. The present secondary analyses from the “Anthocyanins in People at Risk for Dementia Study” were aimed at (I) determining the intervention’s effect on blood-based markers of cardiovascular disease and inflammation and (II) evaluating whether baseline factors such as age, sex, inflammation, or cardiometabolic score may moderate the intervention’s effect on inflammatory status. This study was an ancillary, 24-week randomized, double-blind, placebo-controlled Phase II trial. Sub-sample participants (*n* = 99), aged 60–80 years with mild cognitive impairment or cardiometabolic disorders, were randomized to receive either 320 mg/day of anthocyanins or placebo. The biomarkers analyzed included inflammatory biomarker assessment (IL − 6, IL − 8, IL − 10, IL − 1b, TNF − α, IFN − γ), and C-reactive protein (CRP), as well as albumin, thrombocytes, cholesterol, LDL, HDL, and triglycerides, which were longitudinally compared between both groups. Baseline characteristics were balanced between the groups. ANCOVA analyses reveal 24-week differences favoring the anthocyanin treatment in LDL cholesterol levels (ƞp^2^ = 0.078; *p* = 0.015), cardiometabolic score (ƞp^2^ = 0.073; *p* = 0.021), CRP levels (ƞp^2^ = 0.417; *p* = 0.0001), IL − 6 (ƞp2 = 0.085; *p* = 0.015), IL − 1b (ƞp^2^ = 0.058; *p* = 0.037), and Inflam z-score 5 (ƞp^2^ = 0.059, *p* = 0.004). Moderation analysis demonstrated that the inflammatory score at baseline was a significant predictor of the effect of the intervention on the CRP levels. Anthocyanin supplementation reduces CRP and cardiovascular disease biomarkers in individuals at risk of dementia, especially when there is increased inflammation at baseline. ClinicalTrials.gov study identifier: NCT03419039.

**Supplementary Information:**

The online version contains supplementary material available at 10.1007/s11357-025-01669-8.

## Introduction

With aging, there is a gradual rise in persistent low-grade inflammation, commonly referred to as inflammaging [1, 2]. This state of chronic inflammation is closely linked to the development and progression of age-related diseases, including cardiovascular disorders, cancer, and neurodegenerative conditions [3]. Contributing mechanisms include mitochondrial and cellular dysfunction, oxidative stress, impaired signaling pathways, and apoptosis [3–5]. Additionally, inflammaging may disrupt homeostatic processes and contribute to metabolic dysregulation, leading to endothelial damage, atherosclerosis, hypercoagulation, and neuronal dysfunction and death [6].

This scenario enables the implementation of interventions to mitigate the effects of inflammation and its consequences. One widely used clinical marker of inflammation is C-reactive protein (CRP), which has been linked to adverse health outcomes, particularly in cardiovascular diseases [7]. Modulating inflammation biomarkers could help counteract chronic processes that drive age-related decline, opening avenues for novel public health and pharmacological interventions [3].

Anthocyanins are flavonoids found in dark berries and fruits and are proposed to have health benefits [8]. Their properties include reducing oxidative stress and inflammation, influencing gut microbiota composition, improving endothelial function, lowering LDL cholesterol oxidation, modulating lipid profiles, inhibiting platelet aggregation, and regulating vascular function and blood pressure [9]. Moreover, anthocyanins have shown potential benefits for cognitive health, particularly in enhancing working memory, verbal memory, and executive function, although findings across cognitive domains remain inconsistent [10].

Investigating biomarkers in individuals at risk for age-related diseases provides valuable insights into the role of latent inflammation in adults susceptible to dementia. This approach may contribute to targeted interventions aimed at improving treatment strategies, reducing disease incidence, and promoting healthy aging. The present secondary analyses from the “Anthocyanins in People at Risk for Dementia Study” (ACID) [11] were aimed at: (I) determining the intervention’s effect on blood-based markers of cardiovascular disease and inflammation, and (II) evaluating whether baseline factors such as age, sex, inflammation, or composite cardiometabolic score may moderate the effect of the intervention on the inflammatory status.

## Methods

### Design

This is a secondary analysis of data from a 24-week, randomized, double-blind, placebo-controlled Phase II trial conducted at three centers in Norway between 2018 and 2020, known as the “AnthoCyanins in People at Risk for Dementia study” (ACID). While the primary aim of the trial was to assess cognitive outcomes, the present analysis focuses specifically on the effects of anthocyanin supplementation on biomarkers of inflammation and cardiometabolic health [13]. The study received approval from The Norwegian Regional Ethics Committee (2017/374) and was registered with ClinicalTrials.gov (identifier NCT03419039). All participants provided written informed consent in line with Good Clinical Practice guidelines before enrollment.

### Study population

Participants were recruited through referrals from geriatric, psychiatric, neurology, cardiology, or memory outpatient clinics, as well as from an ongoing cohort study and community and social media advertisements. Initial eligibility was assessed via telephone interviews, followed by in-person evaluations by a research nurse and a study physician. Participants were contacted by telephone after 4 weeks and attended clinic visits at weeks 12 and 24 (final visit). They were instructed to maintain their usual diet and lifestyle throughout the study.

Inclusion criteria targeted individuals aged 60–80 years, including (A) those with mild cognitive impairment (MCI) diagnosed according to the Winblad criteria [12]. A comprehensive neuropsychological battery was administered, comprising the CERAD memory test, Trail Making Tests A and B, the Mini-Mental State Examination (MMSE), and the Informant Questionnaire on Cognitive Decline in the Elderly. To evaluate mood and functional status, the 15-item Geriatric Depression Scale and the Clinical Dementia Rating (CDR) scale were also applied. Informant input for the CDR—typically provided by a partner or adult child—was essential for contextualizing cognitive changes. This group included individuals with and without cardiometabolic disorders (CMDs). Or (B) cognitively healthy individuals with at least two cardiometabolic conditions known to increase the risk of cognitive decline and dementia. These conditions encompassed cerebrovascular disease, stable cardiovascular disease confirmed by angiography, and metabolic disorders such as diabetes mellitus, hypercholesterolemia, hypertension, or overweight, defined as a body mass index (BMI) ≥ 25 [13].

### Randomization, blinding, and packaging

Capsules were identically packaged by the manufacturer, Medox (a product of MedPalett AS, Sandnes, Norway), shipped to the three centers, and dispensed post-allocation. Participants were assigned in a 1:1 ratio to either anthocyanins or placebo using block randomization (block sizes of 4 or 6) within six strata based on two recruitment groups (MCI with or without CMD versus CMD only) and three centers. Anonymous randomization lists were generated by the trial statistician, while the final treatment group allocation was conducted randomly at the medication production site. These randomization lists, containing ID numbers and anonymous treatment arms, were sent to the production site. Participants, study staff, data analysts, and laboratory technicians were blinded throughout the study period. Unblinding occurred after the publication of the final version of the signed statistical analysis plan, database locking, and completion of statistical analyses.

### Diagnostic procedures

A study physician, typically a licensed specialist in psychiatry, geriatrics, or neurology, collected clinical data on medical and psychiatric history as well as sociodemographic information and performed physical examinations.

### Interventional product

The intervention involved two Medox capsules, a standardized nutraceutical product containing 80 mg of naturally purified anthocyanins from bilberry (*Vaccinium myrtillus*) and blackcurrant (*Ribes nigrum*). Each capsule comprised 50% Maltodextrin Glucidex IT 19 and 50% bilberry and blackcurrant extract powder, with 80 mg of anthocyanin citrates as the 3-O-rutinosides of cyanidin and delphinidin, and the 3-O-β-galactopyranosides, 3-O-β-glucopyranosides, and 3-O-α-arabinopyranosides of cyanidin, peonidin, delphinidin, petunidin, and malvidin. Identically appearing placebo capsules (91% maltodextrin and 9% citric acid) were administered twice daily.

The administered doses of the active ingredient were 320 mg of anthocyanins per day, in four capsules divided into two daily doses. The dosage was based on previous clinical trials demonstrating relevant biological changes, good tolerability, and no risk of adverse effects [14, 15]. Participants were asked to return empty blister packages and unused capsules.

### Blood-based biomarkers of cardiovascular disease and inflammation

**Fig. 1:**
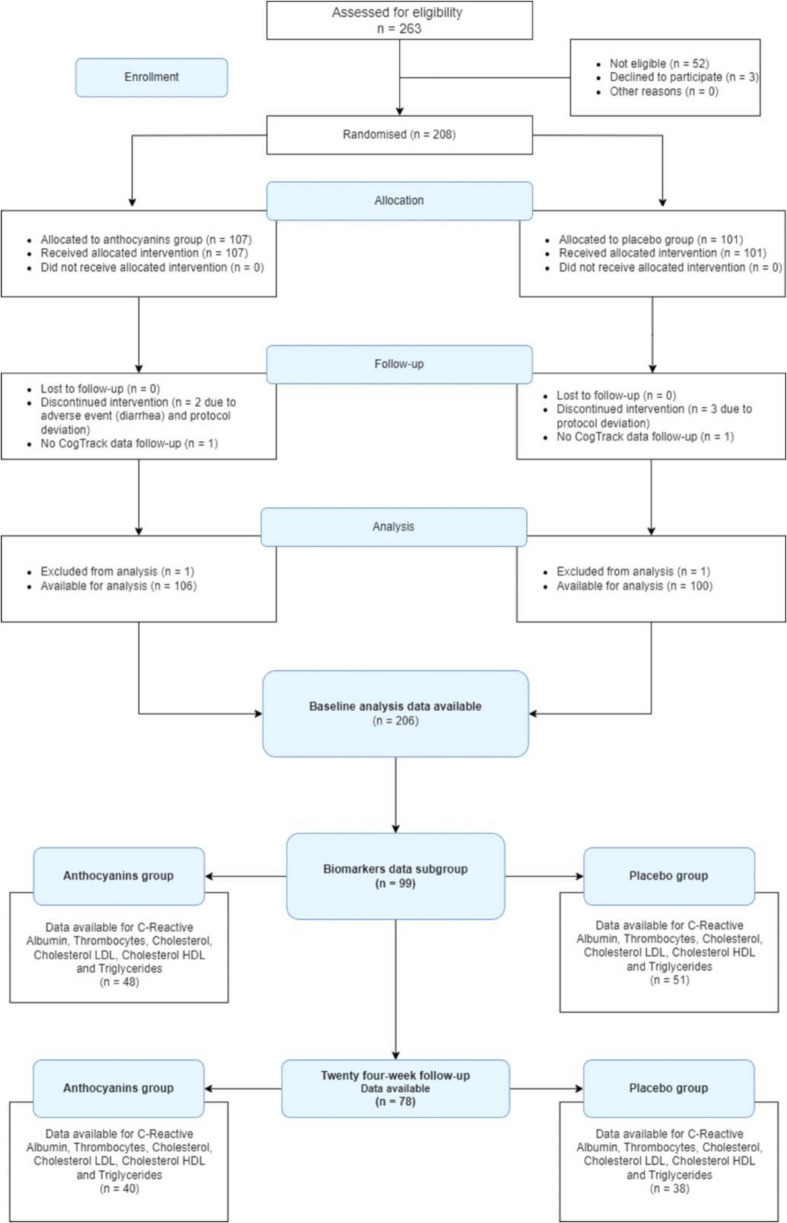
Recruitment and allocation flowchart

Blood samples were collected at weeks 2, 6, 12, and 24, following specific protocols. However, only the baseline and final (24-week) samples were analyzed in this paper. Further details are available in previous publications [13]. Due to logistical constraints and participant availability, complete biomarker analyses were conducted on a subset of participants (see Fig. 1). The cardiovascular biomarkers (CRP, albumin, thrombocytes, glucose, cholesterol, LDL cholesterol, HDL cholesterol and triglycerides) are longitudinally compared between anthocyanins treatment and placebo.

Cardiovascular biomarkers (CRP, albumin, thrombocytes, glucose, cholesterol, LDL cholesterol, HDL cholesterol, and triglycerides) were compared longitudinally between the anthocyanin and placebo groups. To create a composite cardiometabolic score, these markers were standardized as z-scores, summed, and divided by five. This cardiometabolic (CM) z-score included triglycerides, total cholesterol, LDL cholesterol, fasting glucose, and HDL cholesterol (multiplied by − 1 to account for its inverse risk association).

Inflammatory biomarkers—including IL-6, IL-8, IL-10, IL-1β, TNF-α, IFN-γ, and CRP—were measured using the SP-X extended immunoassay kit (Quanterix, Product No. 85–0329) at the Department of Psychiatry and Neurochemistry, University of Gothenburg.

Composite inflammatory scores were generated from log-transformed biomarker concentrations, standardized using the mean and standard deviation of the sample. For the “Inflam z-score 6,” IL − 10 values were multiplied by − 1 to reflect their anti-inflammatory nature. The Inflam z-score 5 included the same biomarkers except IL − 10. Each score was the mean of its components’ standardized values.

Spearman correlations among inflammatory biomarkers and CRP levels showed positive associations across most markers, with the exception of adiponectin, which displayed either negative or non-significant correlations (Supplemental Fig. S1). The Inflam z-score 5 showed a moderate positive correlation with CRP (rs = 0.39, *p* < 0.001). IL − 10 showed inverse correlations, particularly with Inflam z-score 5, IL-8 (rs =  − 0.21, *p* < 0.05), and IFN-γ (rs =  − 0.46, *p* < 0.0001).

### Statistical analysis

A descriptive and exploratory data analysis was conducted by estimating percentages for categorical variables and presenting means with standard deviations, medians with interquartile ranges, and minimum and maximum values for ordinal variables. Subsequently, a group comparison analysis was performed to assess differences between the anthocyanins and placebo groups concerning the variables included in the study at baseline. For categorical variables, Pearson’s chi-square test was employed, while for numeric variables, Student’s *t*-test was used. The diagnostic assessment of the fitted models was conducted by evaluating the normality of residuals and random effects using the Shapiro–Wilk test and visual inspection, assessing collinearity through the variance inflation factor (VIF), and detecting influential outliers using Cook’s distance. These analyses were performed to determine the appropriateness of the models for the data.

A longitudinal data analysis was carried out to determine the effect of anthocyanin treatment vs placebo on each outcome variable using two-way repeated measures (analysis of covariance [ANCOVA]). In this analysis, both groups and assessment time points (baseline and 24 weeks post-intervention) were used as the independent variables, and the baseline scores for the same outcome were the covariates. To quantify the magnitude of the treatment effect on each outcome variable over time, Cohen’s d statistic was calculated. This was obtained by dividing the interaction estimate between treatment and time by the model's residual standard deviation. A significance level of 0.05 was used for hypothesis testing. To determine the effect size of the time × group interaction, the partial eta squared (ηp^2^) was calculated, which was interpreted considering the ηp^2^ values of 0.01, 0.06, and 0.14, which correspond to effect sizes small, moderate, and large, respectively.

**Fig. 2:**
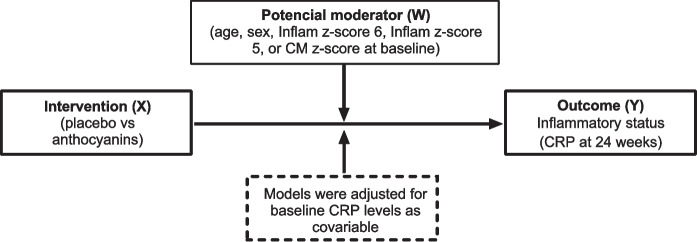
The hypothesized moderation model. Intervention effect on acute-phase protein CRP at 24 weeks, between anthocyanin treatment vs placebo on potential moderator variables at baseline. The potential moderator variables investigated were (age, sex, Inflam z-score 6, Inflam z-score 5 or CM z-score) at baseline study. Each of the potential moderator variables was included in the regression model one at a time, resulting in a total of five models

To investigate the potential influence of moderators and intervention on inflammatory status during the follow-up assessment, we conducted a moderation analysis. This analysis helps to determine whether the values of certain causal variable X (in our study, treatment effect) on outcome Y (in our study, acute-phase protein CRP levels at 24 weeks) is dependent on a moderator variable W (in our study, age, sex, inflammatory score or cardiometabolic score) at baseline study (see Fig. 2). In the model, changes in CRP levels at 24 weeks were regressed on the intervention (anthocyanin treatment vs placebo) while adjusting for baseline CRP levels. Moderating effects were examined by adding the moderator variable and its interaction term with the intervention variable into the regression model. Because the interaction effects were exploratory and intended to generate hypotheses for future research, we used an alpha level of 0.10 for the interaction terms [16]. The results are presented as unstandardized regression coefficients (*β*) and 95% CI.

Models with continuous moderators were estimated using the PROCESS macro-version 4.0 for SPSS. PROCESS uses ordinary least squares (OLS) regression analysis when predicting continuous variables and can be used to visualize interactions using the Johnson–Neyman (J-N) technique [17]. To probe significant interactions, simple slope analysis at low (− 1 SD), average (mean), and high (+ 1 SD) levels of the moderator were used with the J-N technique to assess whether potential moderator values moderated the relationship between the effects of the treatment and CRP levels at 24 weeks, to indicate regions of significance. We used this code and then edited the resulting figure to produce the diagram of the equation (OLS regression) to probe significant interactions [18]. Given that error terms were not normally distributed, a bias-corrected and accelerated (BCa) bootstrap technique with 5000 replicates and resampling of dependent variables with replacement was used as a non-parametric approach. A J-N plot was made by using the coefficients of the regression model in GraphPad Prism, version 8.0 for Windows (GraphPad Software, La Jolla, CA, USA, www.graphpad.com) to visualize the moderated effect of the intervention (anthocyanin treatment vs placebo) on CRP levels at 24 weeks.

## Results

The study design, recruitment process, and sample characteristics are outlined in Fig. 1. Both groups were well-balanced according to sociodemographic and clinical characteristics, with no significant differences observed in lifestyle factors or comorbidities (Table 1).

**Table 1:** Description of the study sample

	Overall	Placebo	Anthocyanins	*p* value
	(*n* = 99)	(*n* = 51)	*(n = 48)*	
Age				
Mean (SD)	69.1 (5.56)	68.8 (5.72)	69.3 (5.43)	0.690
Median [Q1, Q3]	68.0 [64.0, 74.0]	69.0 [63.5, 73.5]	68.0 [65.0, 75.0]	
[MIN, MAX]	[60.0, 79.0]	[60.0, 79.0]	[60.0, 78.0]	
Sex, (%)				
Female	49 (49.5%)	26 (51.0%)	23 (47.9%)	0.918
Male	50 (50.5%)	25 (49.0%)	25 (52.1%)	
Education in years				
Mean (SD)	14.0 (3.24)	13.9 (2.88)	14.1 (3.61)	0.723
Median [Q1, Q3]	14.0 [11.5, 16.5]	14.0 [12.0, 16.0]	14.5 [11.0, 17.0]	
[MIN, MAX]	[8.00, 21.0]	[9.00, 20.0]	[8.00, 21.0]	
Diagnostic group				
CMD	74 (74.7%)	38 (74.5%)	36 (75.0%)	0.999
MCI	25 (25.3%)	13 (25.5%)	12 (25.0%)	

Data presented as *n* (%), mean (standard deviations), median (interquartile range or as minimum and maximum). Abbreviations: MCI mild cognitive impairment, CMD cardiometabolic disorders

Following the intervention, ANCOVA analyses revealed significant differences between the anthocyanin and placebo groups in several biomarkers (Table 2). Specifically, LDL cholesterol levels (ƞp^2^ = 0.078, *p* = 0.015), cardiometabolic score (ƞp^2^ = 0.073, *p* = 0.021), CRP levels (ƞp^2^ = 0.417, *p* < 0.001), IL-6 (ƞp^2^ = 0.085, *p* = 0.015), IL-1β (ƞp^2^ = 0.058, *p* = 0.037), and Inflam z-score 5 (ƞp^2^ = 0.059, *p* = 0.004) showed significant improvements in the anthocyanin group compared to placebo. No other biomarkers exhibited significant differences between the groups.

**Table 2:** Longitudinal adjusted model on the effect of the intervention on the interest variables

Outcome	Baseline (0 week) Mean (SD)		Follow-up (24 weeks) Mean (SD)		Time effect Mean (SEM)		Time × group effect Mean (95%CI)^e^	Effect size (*p*)
	Plac	Anth	Plac	Anth	Plac	Anth	Anth–Plac	ƞp^2^
Albumin	39.35 (2.62)	39.09 (2.66)	38.90 (2.64)	38.83 (2.70)	− 0.14 (0.37)	− 0.15 (0.53)	− 0.017 (− 1.32 to1.28)	0.001 (0.980)
Thrombocytes	245.16 (65.28)	233.23 (55.84)	240.46 (68.44)	222.29 (51.90)	− 3.71 (9.04)	− 22.26 (13.06)	− 18.54 (− 50.25 to 13.16)	0.019 (0.247)
Total cholesterol	4.79 (1.10)	4.79 (1.07)	4.98 (1.32)	4.40 (1.08)	0.11 (0.17)	− 0.44 (0.24)	− 0.55 (− 1.16 to 0.05)	0.044 (0.071)
LDL cholesterol	3.09 (1.08)	2.99 (1.04)	3.34 (1.30)	2.59 (0.98)	0.20 (0.16)	− 0.52 (0.24)	− 0.73 (− 1.31 to − 0.14)	0.078 (0.015)
HDL cholesterol	1.31 (0.30)	1.39 (0.39)	1.31 (0.30)	1.44 (0.41)	− 0.00 (0.04)	0.12 (0.06)	0.13 (− 0.03 to 0.29)	0.032 (0.125)
Triglycerides	1.56 (0.83)	1.61 (0.94)	1.36 (0.76)	1.19 (0.72)	− 0.30 (0.10)	− 0.43 (0.014)	− 0.13 (− 0.49 to 0.23)	0.007 (0.476)
Glucose	6.34 (2.56)	6.23 (1.86)	5.95 (1.25)	6.11 (1.88)	− 0.34 (0.20)	− 0.19 (0.29)	0.15 (− 0.57 to 0.87)	0.002 (0.679)
CM z-score^a^	0.07 (0.58)	− 0.01 (0.51)	0.06 (0.59)	− 2.28 (0.50)	− 0.06 (0.80)	− 0.38 (0.11)	− 0.32 (− 0.59 to − 0.50)	0.073 (0.021)
CRP	1.96 (3.20)	2.18 (2.51)	2.70 (2.52)	0.58 (0.61)	0.88 (0.26)	− 2.56 (0.37)	− 3.45 (− 4.42 to − 2.47)	0.417 (0.0001)
IL-6^b^	0.67 (0.84)	0.35 (1.19)	0.75 (1.07)	0.10 (0.70)	0.06 (0.14)	− 0.54 (0.19)	− 0.60 (− 1.09 to − 0.12)	0.085 (0.015)
IL-8^b^	1.66 (1.21)	1.93 (1.23)	1.71 (1.17)	2.07 (0.94)	− 0.03 (0.15)	0.25 (0.21)	0.29 (− 0.24 to 0.82)	0.016 (0.278)
IL-10^b^	0.71 (0.62)	0.68 (0.79)	0.68 (0.86)	0.43 (0.54)	− 0.00 (0.10)	− 0.25 (0.15)	− 0.25 (− 0.62 to 0.12)	0.023 (0.192)
IL-1b^b^	− 2.69 (1.53)	− 3.02 (1.39)	− 2.72 (1.33)	− 3.35 (0.96)	0.06 (0.17)	− 0.57 (0.24)	− 0.63 (− 1.23 to − 0.03)	0.058 (0.037)
TNF-α^b^	− 0.08 (0.84)	− 0.01 (0.86)	− 0.02 (0.78)	− 0.08 (0.84)	0.01 (0.10)	− 0.08 (0.15)	− 0.09 (− 0.47 to 0.28)	0.003 (0.619)
IFN-γ^b^	− 2.14 (0.98)	− 2.52 (1.04)	− 2.34 (0.86)	− 2.44 (1.16)	− 0.05 (0.13)	− 0.19 (0.19)	− 0.13 (− 0.61 to 0.34)	0.004 (0.573)
Inflam z-score 6^c^	0.08 (0.38)	− 0.06 (0.73)	0.08 (0.41)	− 0.13 (0.40)	0.30 (0.63)	− 0.10 (0.08)	− 1.38 (− 0.34 to 0.74)	0.025 (0.198)
Inflam z-score 5^d^	0.09 (0.40)	− 0.06 (0.72)	0.42 (2.06)	− 0.67 (2.02)	0.35 (0.31)	− 0.71 (0.41)	− 1.07 (− 2.11 to − 0.03)	0.059 (0.044)

*LDL* low-density lipoprotein, *HDL* high-density lipoprotein; units of measure: C-reactive protein (CRP): mg/L, albumin: g/L, thrombocytes: × 10^9/L, cholesterol: mmol/L, cholesterol LDL: mmol/L, cholesterol HDL: mmol/L, Glucose: mmol/L.

^a^Cardiovascular risk markers were converted into standardized z-scores, summed, and divided by five to obtain a cardiometabolic (CM) score. The CM z-score considered the summing z-scores of triglycerides, total cholesterol, (cholesterol HDL multiplying by − 1), cholesterol LDL, and fasting glucose.

^b^Log-transformed biomarker concentrations were used in all analyses.

^c^The Inflammatory scores are derived from log-transformed biomarker concentrations standardized using the mean and standard deviation of our population (z-score); the “Inflam z-score 6” for IL-10 were multiplied by − 1 to account for its anti-inflammatory effect. These z-scores are summed to generate an overall inflammatory score for each individual divided by six.

^d^The inflammatory z-score-5 is composed of the following biomarkers: IL-6, IL-8, IL-1b, TNF-α, and IFN-γ the inflammatory z-score-5 includes the same components as the inflammatory z-score-6, except for the IL-10. These z-scores are summed to generate an overall “Inflam z-score 5” for each individual divided by five.

^e^Adjusted for baseline scores for the same outcome were the covariates. Bold values indicate statistically significant (*p* < 0.05).

**Fig. 3:**
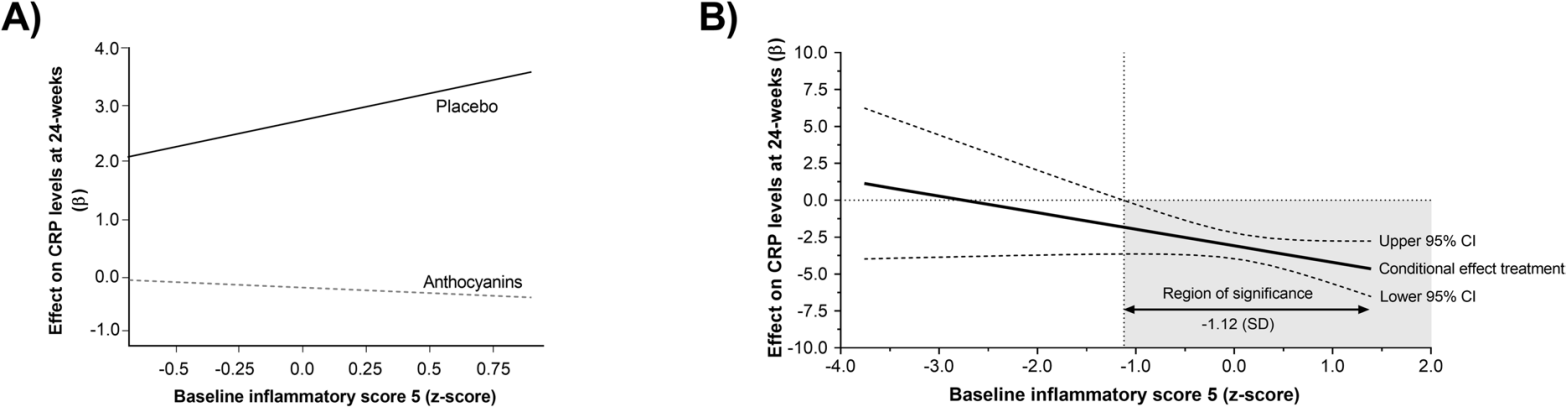
Interaction between treatment with anthocyanins or placebo. A depiction of the interaction between treatment with anthocyanins or placebo and baseline Inflam z-score 5 in the model of CRP at 24-week follow up (**A**) and a Johnson-Neyman plot representing the same interaction with the simple slope (with the 95% confidence intervals) (**B**). Grey area represents the region of significance (*p* < 0.05). Model are adjusted for CRP levels at baseline as covariate

Regression analyses examining potential moderating effects are presented in Table 3. At baseline, the Inflam z-score 5 moderated the effect of the intervention on CRP levels at 24 weeks (*β* = − 1.12, 95% CI − 2.42 to 0.18, *p* = 0.090), but no other potential moderators significantly influenced the effect of the treatment on CRP. Further moderation analysis revealed that individuals with a higher Inflam z-score 5 at baseline experienced a greater reduction in CRP levels following anthocyanin treatment. As illustrated in Fig. 3A, the widening gap between the anthocyanin and placebo groups over the 24 weeks suggests a stronger intervention effect among participants with elevated baseline Inflam z-score 5. Additionally, the Johnson-Neyman (J-N) technique identified a significant intervention effect on CRP levels when the baseline Inflam z-score 5 was below − 1.12 SD, but not when it was at or above this value (Fig. 3B). These findings suggest that baseline Inflam z-score 5 may play a critical role in determining the effectiveness of anthocyanin supplementation in reducing inflammatory markers.

**Table 3:** Results of the analyses examining potential moderators of anthocyanin treatment vs. placebo on CRP levels at 24 weeks

Model	Potential moderator outcome	*β* interaction	95% CI	*p* value
1	Age (years)	0.41	− 0.11 to 0.18	0.604
2	Sex (male/female)	0.06	− 1.55 to 1.32	0.936
3	CM z-score^a^	− 1.08	− 2.69 to 0.52	0.181
4	Inflam z-score 6^b^	− 1.70	− 3.79 to 0.38	0.108
5	Inflam z-score 5^c^	− 1.12	− 2.42 to 0.18	0.090^d^

Legend: ^a^Cardiovascular risk markers were converted into standardized z-scores, summed, and divided by five to obtain a cardiometabolic (CM) score. The CM score considered the summing z-scores of triglycerides, total cholesterol, (cholesterol HDL multiplying by − 1), cholesterol LDL, and fasting glucose

^b^Log-transformed biomarker concentrations were used in all analyses

^c^The Inflammatory scores are derived from log-transformed biomarker concentrations standardized using the mean and standard deviation of our population (z-score); the “Inflam z-score 6” for IL-10 were multiplied by − 1 to account for its anti-inflammatory effect. These z-scores are summed to generate an overall inflammatory score for each individual divided by six. The inflammatory z-score-5 is composed of the following biomarkers: IL-6, IL-8, IL-1b, TNF-α, and IFN-γ; the inflammatory z-score-5 includes the same components as the inflammatory z-score-6, except for the IL-10. These z-scores are summed to generate an overall “Inflam z-score 5” for each individual divided by five

^d^Adjusted for baseline scores for the same outcome were the covariates. Bold values indicate statistical significance for the interaction terms (*p* < 0.10)

## Discussion

This study demonstrates that 24 weeks of anthocyanin supplementation significantly reduced CRP levels, particularly in individuals with pre-existing inflammation, as indicated by an interleukin-based inflammatory score. In contrast, the placebo group experienced increased CRP over the same period. Additionally, anthocyanin intake was associated with improvements in LDL cholesterol, cardiometabolic scores, IL − 6, and IL − 1β, suggesting a broad anti-inflammatory effect. These findings provide pioneering evidence of anthocyanins’ impact on cardiovascular and inflammatory biomarkers in older adults at risk of dementia, supporting further clinical exploration.

CRP is a well-established inflammatory biomarker and risk factor for poor health outcomes [7]. Synthesized by the liver in response to pro-inflammatory cytokines like IL − 6, CRP levels in the blood increase during inflammatory processes. Elevated CRP levels are indicative of systemic inflammation and are associated with a variety of pathological conditions, including infections, chronic inflammatory diseases, and cardiovascular disorders [7]. Its routine clinical measurement allows for the assessment of inflammation severity and monitoring of treatment efficacy [19]. Anthocyanins exert inflammatory effects through multiple pathways, such as the nuclear factor-κ B (NF-kB) and mitogen-activated protein kinase (MAPK) signaling, which downregulates CRP production-related gene expression [9, 20, 21]. Moreover, by activating the nuclear factor erythroid 2-related factor 2 (Nrf2) pathway [22], anthocyanins enhance the activity of endogenous antioxidant enzymes, contributing to neuroprotection and mitigating oxidative stress related to neuroinflammation.

The clinical relevance of reducing CRP to improve health outcomes is increasingly supported by both observational and interventional studies. Elevated CRP levels are strongly associated with higher risk of cardiovascular disease, type 2 diabetes, neurodegeneration, and all-cause mortality [23]. Moreover, trials using statins (e.g., JUPITER trial) and anti-inflammatory agents like canakinumab (CANTOS trial) have shown that lowering CRP independently of lipid levels is linked to reduced cardiovascular events and mortality [23–25]. These findings suggest that CRP may be more than just a marker and could serve as a therapeutic target [23]. Various approaches aimed at lowering CRP include lifestyle interventions, cytokine-targeting therapies, and emerging direct inhibitors. Nutritional compounds like Ginkgo biloba and omega-3 fatty acids have also shown anti-inflammatory, cognitive, and behavioral benefits in clinical trials [26–28]. Also, recent advances — such as small molecule inhibitors, and selective apheresis — have shown promise in reducing CRP, particularly in the context of myocardial infarction [23, 29, 30].

Previous studies on anthocyanins and CRP have yielded mixed results. A meta-analysis [31] of 32 randomized controlled trials reported significant reductions in CRP, IL − 6, TNF − α, ICAM-1, and VCAM − 1, alongside increased adiponectin levels with dietary anthocyanins. In contrast, another meta-analysis [32] found no significant effect on CRP concentrations, with outcomes appearing dependent on the dosage administered. Interestingly, Fallah et al. [31] observed enhanced reductions in CRP, IL − 6, TNF − α, and VCAM − 1 when doses exceeded 300 mg per day, a threshold met in our study with a standard dose of 320 mg of naturally purified anthocyanins from bilberry and blackcurrant. These inconsistencies highlight the potential influence of individual variability in response to anthocyanins [33]. Identifying and targeting the right population for intervention remains a promising avenue for optimizing therapeutic efficacy. While these findings are encouraging, further research is needed to establish robust clinical recommendations and assess the long-term effects of anthocyanins.

While CRP is a widely used marker of systemic inflammation, relying solely on it may not capture the full complexity of inflammatory processes. Other biomarkers such as IL-6 and IL-1β play upstream roles in promoting inflammation and are linked to both peripheral and brain-related inflammatory activity, particularly in the context of aging and cognitive decline [21]. TNF-α is another key cytokine that contributes to vascular damage and neurotoxicity [34]. In parallel, markers like VCAM-1 and ICAM-1 indicate endothelial dysfunction, which is a known contributor to both heart and brain disease [35]. On the other hand, IL-10 is a pleiotropic cytokine that has a fundamental role in modulating inflammation and in maintaining cell homeostasis. It primarily acts as an anti-inflammatory cytokine, protecting the body from an uncontrolled immune response, mostly through the Jak1/Tyk2 and STAT3 signaling pathway [36]. Taking these markers together allows for a more nuanced understanding of inflammation and how anthocyanins might influence it.

While this study focused on a single bioactive component, dietary patterns such as the Mediterranean diet (MD) and Dietary Approaches to Stop Hypertension (DASH) have also demonstrated anti-inflammatory properties. The DASH diet has been associated with significant reductions in high-sensitivity CRP (hs-CRP) levels (mean difference, − 1.01; 95% CI, − 1.64 to − 0.38; I^2^ = 67.7%), particularly in longer-duration trials [37]. Similarly, the PREDIMED study reported reductions in inflammatory cytokines (IL − 1, IL − 6, IL-8, IL − 12p70, CRP, TNF − α) and chemokines (MCP-1, MIP-1β) over short- (e.g., 3 to 12 months) and long-term (≥ 12 months) interventions, with sustained anti-inflammatory effects observed for up to 5 years when the MD was supplemented with olive oil or nuts [38]. These anti-inflammatory effects were sustained for up to 5 years when the MD was supplemented with olive oil or nuts. Notably, our study observed a significant increase in CRP levels in the control group, whereas the anthocyanin-treated group maintained stable levels (Table 3). This supports the notion that anthocyanin-rich berries may contribute to a healthy diet, though their efficacy as a standalone intervention warrants further investigation. Future studies should evaluate the sustained effects of anthocyanins on inflammation over extended periods.

In our analysis, individual biomarkers showed reduced effect sizes, or a loss of statistical significance compared to the composite score, reinforcing its utility. While individual markers such as CRP, IL − 6, or IL − 1b decreased in response to anthocyanins intervention, they can also misclassify others, limiting their standalone utility. In contrast, a composite score integrates complementary information from multiple markers, potentially offering a more reliable classification of inflammatory status (i.e., TNF − α or IL − 8). Notably, although CRP and IL − 6 are closely related, both have been identified as facilitators to the development of neuroinflammation, and, consequently, cognitive decline in older adults [39], underscoring the added value of combining them. These findings suggest that incorporating additional relevant components could further strengthen the robustness and predictive power of the composite inflammatory z-score.

Some limitations of this study should be acknowledged. Firstly, the lack of a comprehensive dietary assessment during the intervention means that unmeasured dietary factors may have influenced anthocyanin absorption. Although participants were advised against major dietary or lifestyle changes, unreported variations could have occurred. Additionally, the study included two distinct cohorts (MCI and CMD), which may have introduced heterogeneity, potentially obscuring additional intervention effects. Furthermore, the effect of anthocyanins on cardiometabolic conditions may differ among individuals with pre-existing cardiovascular risk factors at baseline. Nonetheless, this population diversity could enhance the clinical applicability of our findings across broader groups. Another limitation is the reduced sample size in the final analysis (99 subjects), which could limit statistical power in detecting effects. Importantly, the study’s power calculation was based on cognitive outcomes, not CRP levels, meaning these findings should be interpreted with caution. Another limitation lies in the uncertainty of how accurately circulating inflammatory biomarkers reflect neuroinflammatory processes within the brain. As a result, their association with cognitive decline in older adults may not fully capture the complexity or specificity of localized inflammation in neural tissue. Future trials with larger cohorts are warranted to validate these observations.

Our study has several key strengths. Firstly, it utilized meticulously standardized purified anthocyanins sourced from bilberry and blackcurrant, enhancing the precision of evaluating the biological effects of the phenolic compounds studied and eliminating potential interference from other bioactive food components and food matrices. Additionally, the study involved a homogeneous population from Norway with similar habits and diets. Moreover, significant effects were observed within a relatively short intervention period.

In conclusion, anthocyanin supplementation was associated with a significant decrease in CRP levels over 24 weeks, in contrast to the significant increase observed in the placebo group. In addition, there was also a benefit in LDL cholesterol levels, cardiometabolic scores, and IL − 6 and IL − 1β. Finally, the effect of anthocyanin on CRP levels was influenced by elevated inflammation at baseline. Incorporating anthocyanins into dietary strategies could be a useful approach for managing chronic inflammation-related conditions and may complement existing therapeutic options.

## Supplementary Information

Below is the link to the electronic supplementary material.

Supplementary file1 (PDF 218 KB)

## References

[1] Fulop T, Larbi A, Dupuis G, Le Page A, Frost EH, Cohen AA, et al. Immunosenescence and inflamm-aging as two sides of the same coin: friends or foes? Front Immunol. 2017;8:1960. 29375577 10.3389/fimmu.2017.01960 PMC5767595

[2] Li X, Li C, Zhang W, Wang Y, Qian P, Huang H. Inflammation and aging: signaling pathways and intervention therapies. Signal Transduct Target Ther. 2023;8(1):239. 37291105 10.1038/s41392-023-01502-8 PMC10248351

[3] Chitnis T, Weiner HL. CNS inflammation and neurodegeneration. J Clin Invest. 2017;127(10):3577–87. 28872464 10.1172/JCI90609 PMC5617655

[4] Ben Haim L, Rowitch DH. Functional diversity of astrocytes in neural circuit regulation. Nat Rev Neurosci. 2017;18(1):31–41. 27904142 10.1038/nrn.2016.159

[5] Liddelow SA, Guttenplan KA, Clarke LE, Bennett FC, Bohlen CJ, Schirmer L, et al. Neurotoxic reactive astrocytes are induced by activated microglia. Nature. 2017;541(7638):481–7. 28099414 10.1038/nature21029 PMC5404890

[6] Heneka MT, Carson MJ, El Khoury J, Landreth GE, Brosseron F, Feinstein DL, et al. Neuroinflammation in Alzheimer’s disease. Lancet Neurol. 2015;14(4):388–405. 25792098 10.1016/S1474-4422(15)70016-5 PMC5909703

[7] Amezcua-Castillo E, González-Pacheco H, Sáenz-San Martín A, Méndez-Ocampo P, Gutierrez-Moctezuma I, Massó F, et al. C-Reactive protein: the quintessential marker of systemic inflammation in coronary artery disease-advancing toward precision medicine. Biomedicines. 2023;11(9):2444. 10.3390/biomedicines11092444 PMC10525787 37760885

[8] Lila MA. Anthocyanins and human health: an in vitro investigative approach. J Biomed Biotechnol. 2004;2004(5):306–13. 15577194 10.1155/S111072430440401X PMC1082894

[9] Hair R, Sakaki JR, Chun OK. Anthocyanins, microbiome and health benefits in aging. Molecules. 2021;26(3):537. 10.3390/molecules26030537 PMC7864342 33494165

[10] Ellis LR, Boesch C, Dye L. Effects of anthocyanins on cognition and vascular function: a systematic review. Mol Nutr Food Res. 2024;68(13):e2300502. 38961529 10.1002/mnfr.202300502

[11] Aarsland D, Khalifa K, Bergland AK, Soennesyn H, Oppedal K, Holteng LBA, et al. A randomised placebo-controlled study of purified anthocyanins on cognition in individuals at increased risk for dementia. Am J Geriatr Psychiatry. 2023;31(2):141–51. 36372613 10.1016/j.jagp.2022.10.002

[12] Bondi MW, Edmonds EC, Jak AJ, Clark LR, Delano-Wood L, McDonald CR, et al. Neuropsychological criteria for mild cognitive impairment improves diagnostic precision, biomarker associations, and progression rates. J Alzheimers Dis. 2014;42(1):275–89. 24844687 10.3233/JAD-140276 PMC4133291

[13] Khalifa K, Bergland AK, Soennesyn H, Oppedal K, Oesterhus R, Dalen I, et al. Effects of purified anthocyanins in people at risk for dementia: study protocol for a phase II randomized controlled trial. Front Neurol. 2020;11:916. 32982933 10.3389/fneur.2020.00916 PMC7492209

[14] Bergland AK, Soennesyn H, Dalen I, Rodriguez-Mateos A, Berge RK, Giil LM, et al. Effects of anthocyanin supplementation on serum lipids, glucose, markers of inflammation and cognition in adults with increased risk of dementia - a pilot study. Front Genet. 2019;10:536. 31244884 10.3389/fgene.2019.00536 PMC6581024

[15] Zhang H, Xu Z, Zhao H, Wang X, Pang J, Li Q, et al. Anthocyanin supplementation improves anti-oxidative and anti-inflammatory capacity in a dose-response manner in subjects with dyslipidemia. Redox Biol. 2020;32:101474. 32179241 10.1016/j.redox.2020.101474 PMC7078384

[16] Durand CP. Does raising type 1 error rate improve power to detect interactions in linear regression models? A simulation study. PLoS ONE. 2013;8(8): e71079. 23976980 10.1371/journal.pone.0071079 PMC3745431

[17] Lin H. Probing two-way moderation effects: a review of software to easily plot Johnson-Neyman figures. Struct Equ Modeling. 2020;27(3):494–502.

[18] Hayes AF, Rockwood NJ. Regression-based statistical mediation and moderation analysis in clinical research: observations, recommendations, and implementation. Behav Res Ther. 2017;98:39–57. 27865431 10.1016/j.brat.2016.11.001

[19] Sproston NR, Ashworth JJ. Role of C-reactive protein at sites of inflammation and infection. Front Immunol. 2018;9:754. 29706967 10.3389/fimmu.2018.00754 PMC5908901

[20] Ma Z, Du B, Li J, Yang Y, Zhu F. An insight into anti-inflammatory activities and inflammation related diseases of anthocyanins: a review of both in vivo and in vitro investigations. Int J Mol Sci. 2021;22(20):11076. 10.3390/ijms222011076 PMC8540239 34681733

[21] Zhang W, Xiao D, Mao Q, Xia H. Role of neuroinflammation in neurodegeneration development. Signal Transduct Target Ther. 2023;8(1):267. 37433768 10.1038/s41392-023-01486-5 PMC10336149

[22] Moratilla-Rivera I, Sánchez M, Valdés-González JA, Gómez-Serranillos MP. Natural products as modulators of Nrf2 signaling pathway in neuroprotection. Int J Mol Sci. 2023;24(4):3748. 10.3390/ijms24043748 PMC9967135 36835155

[23] Jimenez RV, Szalai AJ. Therapeutic lowering of C-reactive protein. Front Immunol. 2020;11: 619564. 33633738 10.3389/fimmu.2020.619564 PMC7901964

[24] Ridker PM, Danielson E, Fonseca FA, Genest J, Gotto AM Jr, Kastelein JJ, et al. Rosuvastatin to prevent vascular events in men and women with elevated C-reactive protein. N Engl J Med. 2008;359(21):2195–207. 18997196 10.1056/NEJMoa0807646

[25] Ridker PM, Everett BM, Thuren T, MacFadyen JG, Chang WH, Ballantyne C, et al. Antiinflammatory therapy with canakinumab for atherosclerotic disease. N Engl J Med. 2017;377(12):1119–31. 28845751 10.1056/NEJMoa1707914

[26] Elisia I, Yeung M, Kowalski S, Wong J, Rafiei H, Dyer RA, et al. Omega 3 supplementation reduces C-reactive protein, prostaglandin E(2) and the granulocyte/lymphocyte ratio in heavy smokers: an open-label randomized crossover trial. Front Nutr. 2022;9:1051418. 36532545 10.3389/fnut.2022.1051418 PMC9751896

[27] Mousavi SN, Hosseinikia M, Yousefi Rad E, Saboori S. Beneficial effects of Ginkgo biloba leaf extract on inflammatory markers: a systematic review and meta-analysis of the clinical trials. Phytother Res. 2022;36(9):3459–69. 35781715 10.1002/ptr.7544

[28] Pagotto GLO, Santos LMOd, Osman N, Lamas CB, Laurindo LF, Pomini KT, et al. Ginkgo biloba: a leaf of hope in the fight against Alzheimer’s dementia: clinical trial systematic review. Antioxidants. 2024;13(6):651. 10.3390/antiox13060651 PMC11201198 38929090

[29] Jones NR, Pegues MA, McCrory MA, Singleton W, Bethune C, Baker BF, et al. A selective inhibitor of human C-reactive protein translation is efficacious in vitro and in C-reactive protein transgenic mice and humans. Mol Ther Nucleic Acids. 2012;1(11):e52. 23629027 10.1038/mtna.2012.44 PMC3511672

[30] Torzewski J, Heigl F, Zimmermann O, Wagner F, Schumann C, Hettich R, et al. First-in-man: case report of selective C-reactive protein apheresis in a patient with SARS-CoV-2 infection. Am J Case Rep. 2020;14(21):e925020. 10.12659/AJCR.925020 PMC7377527 32661220

[31] Fallah AA, Sarmast E, Fatehi P, Jafari T. Impact of dietary anthocyanins on systemic and vascular inflammation: systematic review and meta-analysis on randomised clinical trials. Food Chem Toxicol. 2020;135:110922. 31669599 10.1016/j.fct.2019.110922

[32] Sangsefidi ZS, Hasanizadeh S, Hosseinzadeh M. Effect of purified anthocyanins or anthocyanin-rich extracts on C-reactive protein levels: a systematic review and meta-analysis of randomised clinical trials. Br J Nutr. 2018;120(12):1406–14. 30375293 10.1017/S0007114518002957

[33] Feng RC, Dong YH, Hong XL, Su Y, Wu XV. Effects of anthocyanin-rich supplementation on cognition of the cognitively healthy middle-aged and older adults: a systematic review and meta-analysis of randomized controlled trials. Nutr Rev. 2023;81(3):287–303. 35960187 10.1093/nutrit/nuac055

[34] Gonzalez CN. Role of tumor necrosis factor-alpha in the central nervous system: a focus on autoimmune disorders. Front Immunol. 2023;14:1213448. 37483590 10.3389/fimmu.2023.1213448 PMC10360935

[35] Singh V, Kaur R, Kumari P, Pasricha C, Singh R. ICAM-1 and VCAM-1: Gatekeepers in various inflammatory and cardiovascular disorders. Clin Chim Acta. 2023;1(548):117487. 10.1016/j.cca.2023.117487 37442359

[36] Lei X, Qiu S, Yang G, Wu Q. Adiponectin and metabolic cardiovascular diseases: therapeutic opportunities and challenges. Genes Dis. 2023;10(4):1525–36. 37397515 10.1016/j.gendis.2022.10.018 PMC10311114

[37] Soltani S, Chitsazi MJ, Salehi-Abargouei A. The effect of dietary approaches to stop hypertension (DASH) on serum inflammatory markers: a systematic review and meta-analysis of randomized trials. Clin Nutr. 2018;37(2):542–50. 28302405 10.1016/j.clnu.2017.02.018

[38] Casas R, Sacanella E, Urpí-Sardà M, Corella D, Castañer O, Lamuela-Raventos RM, et al. Long-term immunomodulatory effects of a Mediterranean diet in adults at high risk of cardiovascular disease in the PREvención con DIeta MEDiterránea (PREDIMED) randomized controlled trial. J Nutr. 2016;146(9):1684–93. 27440261 10.3945/jn.115.229476

[39] Feng L, Wang Y, Zeng D, Wang M, Duan X. Predictors of cognitive decline in older individuals without dementia: an updated meta-analysis. Ann Clin Transl Neurol. 2023;10(4):497–506. 36705073 10.1002/acn3.51740 PMC10109353

