# Supplementary material for: Anthocyanin supplementation in adults at risk for dementia: a randomized controlled trial on its cardiometabolic and anti-inflammatory biomarker effects
Source: GeroScience. 2025 May 2;48(1):563–76. doi: 10.1007/s11357-025-01669-8 (PMC12972345; doi:10.1007/s11357-025-01669-8)
Supplement: Supplementary file 1 — Supplementary file1 (PDF 218 KB) [file 11357_2025_1669_MOESM1_ESM.pdf]

Suplemental files

Fig. 1 Spearman correlations of individual log-transformed inflammatory biomarkers, inflam z-score 6 and inflam z-score 5.

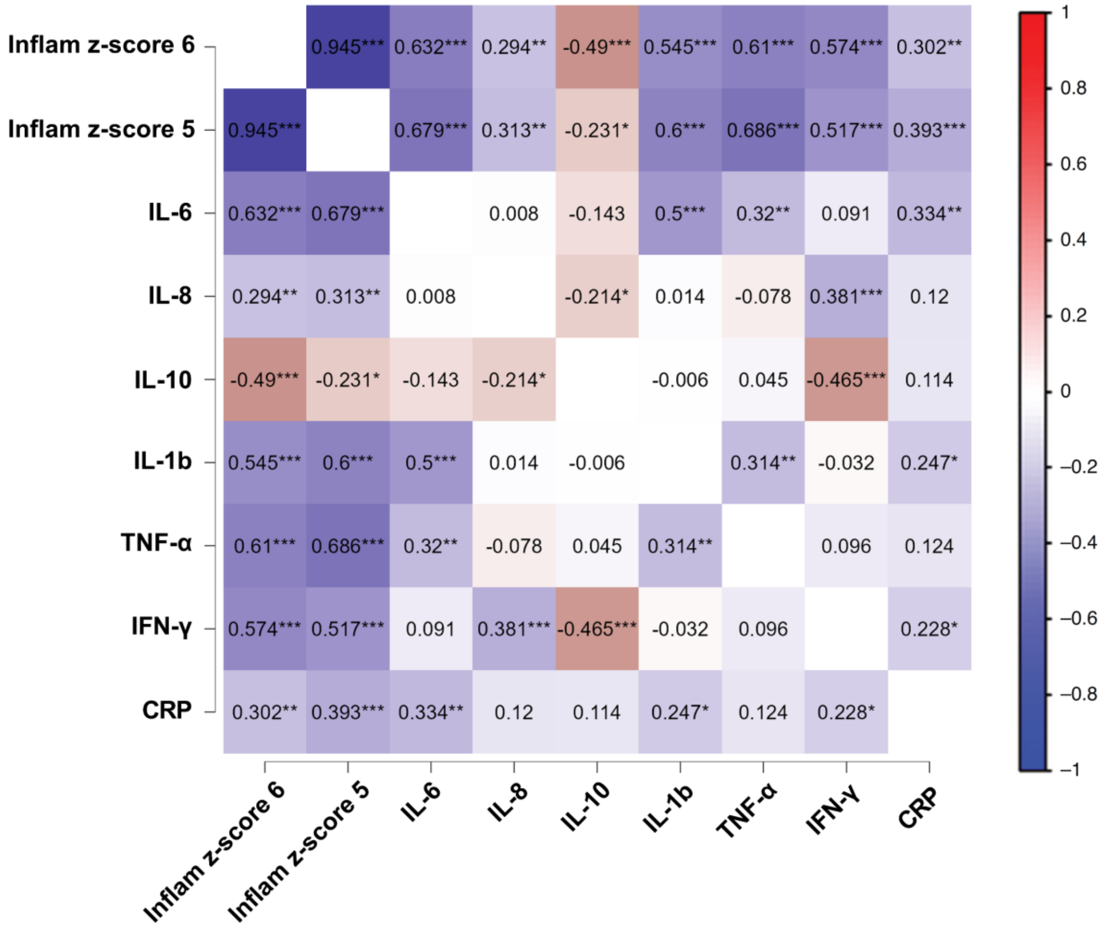

Legend: Asterisks indicate statistically significant correlations (*p < 0.05, p < 0.001**, p < 0.0001***).
